# SAFE (Subarachnoid-Alternative Anaesthesia for Endoprosthesis): A Motor-Sparing and Opioid-Sparing Anesthetic Technique for Hip Fracture Surgery

**DOI:** 10.3390/jcm15103808

**Published:** 2026-05-15

**Authors:** Romualdo Del Buono, Raffaella Barretta, Paola Marsico, Chiara Palermo, Fabio Costa, Giuseppe Pascarella, Giorgio Ranieri, Andrea Tognù

**Affiliations:** 1 Anesthesiology and Intensive Care Unit, ASST Gaetano Pini-CTO, 20122 Milan, Italy; barrettaraffaella@gmail.com (R.B.); chiara.palermo11@gmail.com (C.P.); andrea.tognu@asst-pini-cto.it (A.T.); 2 Anesthesiology and Intensive Care Unit, Valle d’Itria Hospital, 74015 Martina Franca, Italy; mars.paola19@gmail.com; 3 Anesthesiology and Intensive Care Unit, Fondazione Policlinico Universitario Campus Bio-Medico, 00128 Rome, Italy; f.costa@policlinicocampus.it (F.C.); g.pascarella@policlinicocampus.it (G.P.); 4 Anesthesiology and Intensive Care Unit, Isola Tiberina Hospital—Gemelli Isola, 00186 Rome, Italy; gioranieri84@gmail.com

**Keywords:** nerve block, arthroplasty, replacement, hip, frail elderly, anesthesia, conduction, enhanced recovery after surgery, pain, postoperative, pericapsular nerve group block, posterior pericapsular nerve group block, local infiltration analgesia, opioid-sparing anesthesia

## Abstract

**Background**: Anesthetizing frail patients for hip surgery is challenging; spinal (SA) and general anesthesia (GA) often cause hemodynamic instability. Traditional nerve blocks provide analgesia but rarely complete surgical anesthesia without motor block. We evaluate the clinical feasibility of the SAFE (Subarachnoid-alternative Anaesthesia For Endoprosthesis) protocol—combining Anterior Pericapsular Nerve Group (A-PENG), POsterior pericapsular Nerve Group (PONG), and Local Infiltration Analgesia (LIA) under intravenous sedation—as a primary anesthetic preserving motor function and avoiding SA/GA. **Methods**: This single-center retrospective series analyzed patients undergoing elective or trauma-related hip surgery using the SAFE protocol between September 2022 and April 2026. The primary outcome was success rate (completion without SA/GA conversion). Secondary outcomes included procedural timings, recovery room (RR) transit, and motor preservation. Variables are reported as medians [IQR]. **Results**: We included 48 patients (median age 83.5 years [IQR: 68.7–87.2]; 66.7% female) undergoing hip hemiarthroplasty (*n* = 28) or total hip arthroplasty (*n* = 20). The success rate was 100%, without SA/GA conversion or advanced airway management. Median anesthetic preparation and surgical durations were 55 [IQR: 50–76.2] and 85 min [IQR: 74–110], respectively. RR transit times (recorded for 35 patients) were brief (40 min [IQR: 34.0–67.5]). Crucially, lower-limb motor capacity was preserved in 100% of cases. The technique also proved opioid-sparing, substantially reducing postoperative opioid consumption. **Conclusions**: The SAFE protocol is a clinically feasible primary anesthetic strategy for hip surgery. By preserving motor function and enabling rapid fast-tracking, it aligns with ERAS pathways, offering a promising alternative to conventional anesthesia for elective and frail trauma patients. Randomized controlled trials are warranted to validate these outcomes.

## 1. Introduction

Hip fractures in the elderly are a severe event with a significant impact on public health and quality of life. Current guidelines recommend that surgery should be performed as early as possible, ideally within 48 h of admission, to reduce mortality, minimize prolonged suffering, and prevent complications such as pressure ulcers and thromboembolic events [1,2].

The aging population often presents with multiple comorbidities (frailty), which inherently increases perioperative risk [3,4]. The choice of anesthetic technique plays a crucial role in patient outcomes. While Spinal Anesthesia (SA) is widely utilized and considered a first-line option in Europe to avoid General Anesthesia (GA) complications, it still carries risks such as severe hypotension and bradycardia. Conversely, GA in the elderly is associated with hemodynamic depression or respiratory issues. In both cases, there is a chance of postoperative delirium, especially when deep sedation or excessive opioid use is involved [5,6].

To mitigate these risks, regional anesthesia (RA) techniques have gained traction as part of a multimodal analgesic approach. Traditional blocks, such as the Fascia Iliaca Compartment Block (FICB) or femoral nerve block (FNB), provide significant analgesia but inevitably cause quadriceps weakness, hindering the early mobilization goals of Enhanced Recovery After Surgery (ERAS) protocols [7,8,9].

The Pericapsular Nerve Group (PENG) block, introduced by Girón-Arango et al. in 2018 [10], offers an effective motor-sparing alternative by targeting the articular branches of the femoral, obturator, and accessory obturator nerves supplying the anterior hip capsule. However, the PENG block alone is insufficient for surgical anesthesia because it does not cover the overlying tissues nor the posterior capsule, the latter being innervated by the nerve to the quadratus femoris and superior gluteal nerve mainly.

To overcome this limitation, we developed the SAFE (Subarachnoid-alternative Anaesthesia For Endoprosthesis) technique and started using it in selected patients. This novel strategy combines an Anterior modified PENG (A-PENG) block, a POsterior pericapsular Nerve Group (PONG) block, and Local Infiltration Analgesia (LIA) under light sedation in spontaneously breathing patients. The primary aim of this retrospective observational study was to evaluate the efficacy and safety of the SAFE technique as the sole anesthetic modality for hip surgeries (hip hemiarthroplasty and total hip replacements) especially after hip fractures, avoiding the need for SA or GA.

## 2. Materials and Methods

### 2.1. Study Design and Participants

This is a retrospective, observational, descriptive study involving adult patients who underwent hip hemiarthroplasty for hip fractures and total hip replacements at the Anesthesia and Intensive Care Unit of ASST Pini-CTO Hospital (Milan, Italy) between September 2022 and April 2026.

The cohort consisted of a convenience sample of patients. Subjects were not strictly consecutive; they were included if they received the SAFE protocol, which was dependent on the clinical shift availability of a senior anesthesiologist skilled in this specific advanced regional technique.

The hospital electronic database (Ormaweb, Dedalus SpA, Milan, Italy) was initially queried to retrieve all surgical procedures performed during the study period. From this general cohort, patients were selected using International Classification of Diseases (ICD) codes corresponding to either a diagnosis or a surgical procedure specifically related to hip hemiarthroplasty or total hip replacement. Subsequently, a secondary filter was applied to isolate cases where the recorded anesthetic technique was ‘local anesthesia’ combined with ‘sedation’. Finally, all individual electronic records were manually audited to verify the clinical course and to identify any intraoperative conversion to alternative anesthetic techniques and to confirm or exclude the execution of the technique A-PENG/PONG/LIA.

Inclusion criteria:

- Age >18 years.
- Intervention: hip hemiarthroplasty; total hip replacement.
- Anesthesia type: local anesthesia (= A-PENG + PONG + LIA), sedation.
- ASA physical status I–IV.

Given the retrospective nature of the database query, the ASA score was utilized as the standardized surrogate marker for patient comorbidities and frailty.

Exclusion criteria:

- Pre-existing neurological deficits of the affected limb.
- Absolute contraindications to peripheral nerve blocks.
- Incomplete anesthesia records.
- Other regional techniques performed or general anesthesia as the main technique.

#### Perioperative Management

The following sections describe the perioperative management of all patients that underwent this procedure.

All ultrasound-guided blocks were performed by, or under the direct clinical supervision of, a senior anesthesiologist with extensive expertise in regional anesthesia.

### 2.2. Preoperative Anesthetic Management

In the preoperative room, standard monitoring (ECG, HR, NIBP, SpO2) was applied, and peripheral venous access was secured. Patients received premedication with midazolam (1–5 mg) and fentanyl (50–100 µg) prior to block execution.

The SAFE protocol is executed in a precise, stepwise manner using three prepared syringes:

– Syringe 1 (A-PENG block): 10 mL Mepivacaine 2% with epinephrine 1:200,000 + 10 mL Ropivacaine 0.75% + 2 mg dexamethasone.
– Syringe 2 (LIA): 5 mL Mepivacaine 2% with epinephrine 1:200,000 + 5 mL Ropivacaine 0.75% + 1 mg dexamethasone + 10 mL NaCl 0.9%.
– Syringe 3 (PONG block): 5 mL Mepivacaine 2% with epinephrine 1:200,000 + 5 mL Ropivacaine 0.75% + 1 mg dexamethasone.

#### 2.2.1. STEP 1: A-PENG Block

**Figure 1:**
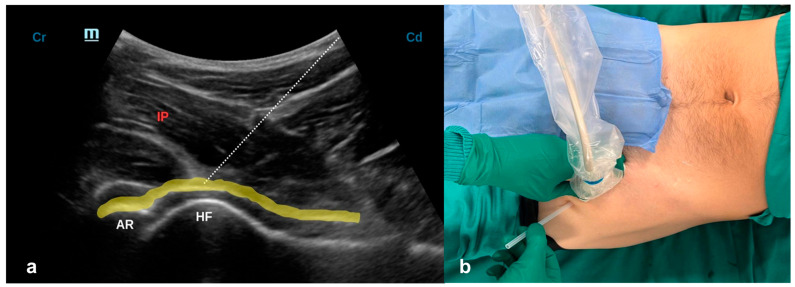
(**a**) Ultrasound scan for A-Peng block. The needle tip (dashed line) is advanced toward the pericapsular plane. The yellow line indicates the ideal distribution of the LA along the entire pericapsular plane, deep to the IP. Cr = cranial; Cd = caudal; AR = acetabular rim; HF = femoral head; IP = iliopsoas muscle. (**b**) Probe positioning for the A-Peng block. With the patient in the supine position, the convex probe is placed in a plane parallel to the long axis of the femoral neck. An echogenic needle is inserted in-plane, using a caudo-cranial approach, from lateral to medial.

With the patient in a supine position, a low-frequency convex probe (2–5 MHz) is placed parallel to the long axis of the femoral neck [11]. This view allows visualization of the acetabulum, femoral neck and head, the hyperechoic capsular plane, and the overlying iliopsoas muscle. An 80–100 mm echogenic needle (22G) is advanced in-plane from lateral to medial, caudo-cranially, targeting the pericapsular plane just caudal to the acetabular rim. Once correctly positioned without breaching the capsule, 20 mL of the local anesthetic (LA) mixture (Syringe 1: 10 mL Mepivacaine 2% with epinephrine 1:200,000 + 10 mL Ropivacaine 0.75% + 2 mg dexamethasone.) is injected (Figure 1).

If properly injected, the LA diffuses in a dome-shaped form along the anterior capsule.

#### 2.2.2. STEP 2: Local Infiltration Analgesia (LIA)

Following the marking of the surgical incision line by the orthopedic surgeon, 10 mL of the diluted LA mixture (Syringe 2: 5 mL Mepivacaine 2% with epinephrine 1:200,000 + 5 mL Ropivacaine 0.75% + 1 mg dexamethasone + 10 mL NaCl 0.9%) is infiltrated along the trajectory of incision in asepsis. The remaining 10 mL is injected subcutaneously in a fan-shaped form from the middle of the incision towards the hip. For the lateral and posterolateral approaches, few mL is eventually injected with the needle tip in contact with the supero-postero-lateral surface of the greater throchanter, during the fan-shaped injection.

#### 2.2.3. STEP 3: PONG Block

**Figure 2:**
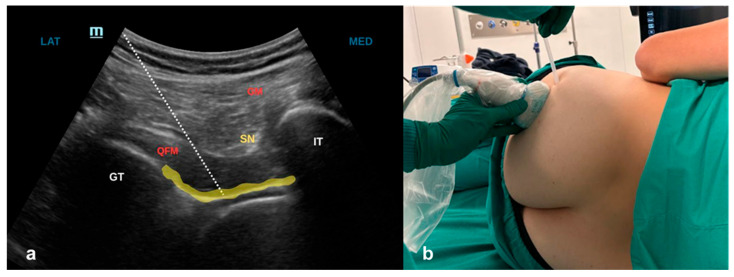
(**a**) Ultrasound scan for PONG block. The needle tip (dashed line) is advanced toward the pericapsular plane. The yellow line indicates the ideal distribution of the LA beneath the QFM. LAT = lateral; MED = medial; GT = greater trochanter; IT = Ischial tuberosity; SN = sciatic nerve; GM = gluteus maximus muscle; QFM = quadratus femoris muscle. (**b**) Probe positioning for the PONG block. In the lateral position with the fractured side up, the convex probe is placed along the line between GT and IT. An echogenic needle is inserted in-plane, using a lateral-medial approach.

The patient is then rotated to the lateral decubitus position with the fractured side up. A low-frequency convex probe is placed transversally between the greater trochanter and the ischial tuberosity. The gluteus maximus, sciatic nerve, and quadratus femoris muscle are identified. The needle is inserted in-plane from lateral to medial, carefully avoiding the sciatic nerve. After negative aspiration, 10 mL of LA (Syringe 3: 5 mL Mepivacaine 2% with epinephrine 1:200,000 + 5 mL Ropivacaine 0.75% + 1 mg dexamethasone) is injected beneath the quadratus femoris muscle [12] (Figure 2).

### 2.3. Intraoperative Management

Patients are transferred to the operating room and placed under light sedation using Propofol via Target Controlled Infusion (TCI), titrating the effect-site concentration (Ce 1.0–3.0 µg/mL). The depth of sedation is adapted to ensure comfort and spontaneous breathing [13].

### 2.4. Postoperative Analgesia and Assessment

In the recovery room, patients are assessed for pain needing rescue analgesics and immediate motor function. 

Motor preservation was clinically assessed by the attending anesthesiologist by requesting the patient to perform active flexion and extension of the hip, knee, and ankle of the operated limb. The Bromage score was not applied because this scale cannot selectively assess an eventual motor block of the sciatic or femoral nerves. We acknowledge that the lack of a formalized scoring system in the database is a constraint of this retrospective study.

On the surgical ward, if there are no patient-specific contraindications, the institutional standard-of-care analgesic protocol was applied, consisting of scheduled Paracetamol 1000 mg and Ketorolac 30 mg (or Ibuprofen 600 mg) every 8 h. If the patient reported a Numeric Rating Scale (NRS) > 6, specific rescue opioids were prescribed: either intravenous Tramadol 50–100 mg or subcutaneous Morphine 2 mg, up to a maximum of one dose every 8 h. Intraoperative and Post-Anesthesia Care Unit (PACU) opioid administrations were extracted directly from the anesthesia records.

Finally, regarding key Enhanced Recovery After Surgery (ERAS) outcomes—specifically the exact time to first mobilization, incidence of postoperative delirium, total length of hospital stay, and final discharge destination—these variables were not systematically coded in the primary electronic anesthesia database. Since our methodology relied on the extraction of digital perioperative records rather than manual reviews of individual ward medical charts, these specific parameters could not be reliably retrieved and are therefore not included in the present analysis.

### 2.5. Data Collection and Statistical Analysis

Clinical and operational data were retrospectively extracted from the institutional electronic operating room management system (Ormaweb, Dedalus SpA) utilizing the dedicated intervention data extraction module. Prior to any analytical procedure, the dataset was rigorously anonymized by the investigators by removing all direct personal identifiers (e.g., names and exact dates of birth) in strict compliance with current data protection regulations, ensuring a fully pseudonymous dataset.

Categorical variables are expressed as absolute frequencies and percentages. Given the intrinsic non-normal distribution of clinical and operational time intervals in surgical settings, continuous variables (such as patient age, anesthetic preparation time, and surgical duration) were analyzed non-parametrically and are reported directly as medians and interquartile ranges (IQRs). Missing data for specific variables (e.g., ASA physical status score) were handled by excluding the missing cases from the specific denominator, without applying any data imputation techniques.

Database filtering, data transformation, and all descriptive statistical analyses were performed using custom Python scripts (version 3.10, Python Software Foundation, Wilmington, DE, USA), utilizing *pandas* and *numpy* libraries, systematically generated and executed via the Gemini large language model environment (version 3.1 PRO, Google LLC, Mountain View, CA, USA).

During the preparation of this work, the authors used the Gemini large language model (Google LLC) to assist in generating the Python scripts used for data extraction and for English language editing. After using this tool, the authors rigorously reviewed and validated the code and the text and take full responsibility for the final content of the publication.

## 3. Results

### 3.1. Demographic and Clinical Characteristics

**Figure 3:**
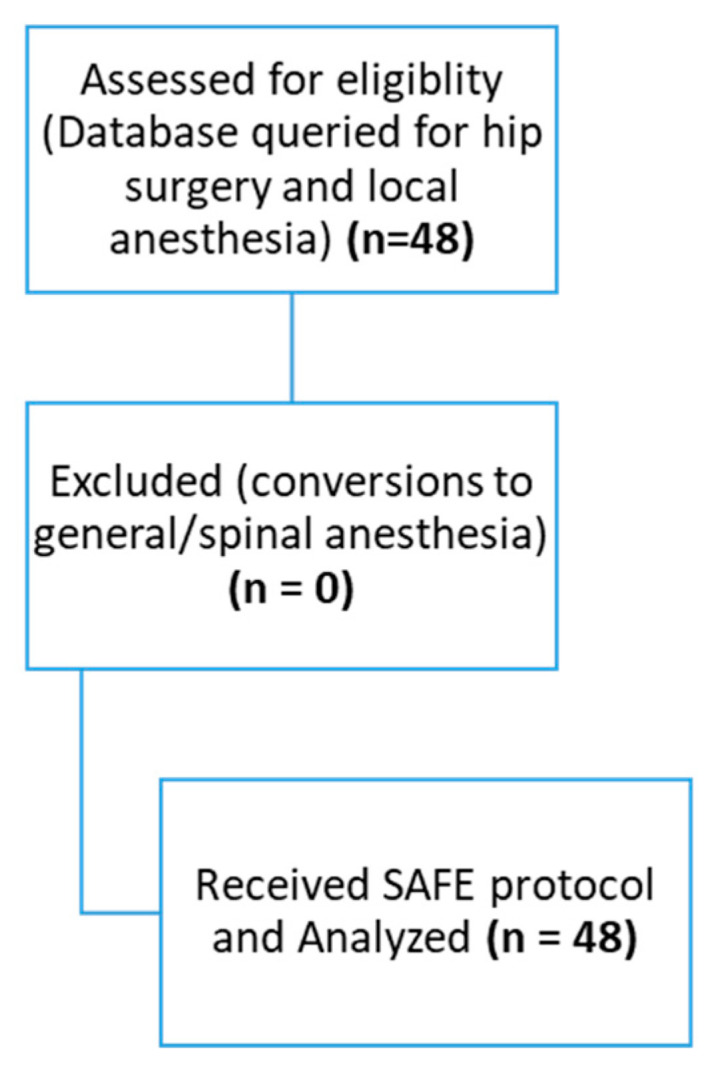
Patient flow diagram. A total of 48 patients were screened and successfully received the SAFE protocol; no patients were excluded or required conversion to alternative anesthetic techniques.

The electronic anesthesia database was queried for patients undergoing hip hemiarthroplasty or total hip replacement under a combination of local anesthesia and sedation. A total of 48 patients matching these criteria were identified. Since no patients required conversion to general or neuraxial anesthesia, all 48 subjects successfully received the SAFE protocol as planned. Consequently, no patients were excluded, and all 48 subjects were included in the final clinical analysis. (Figure 3).

The procedures were performed over a 5-year period, demonstrating a progressive institutional adoption of this anesthetic technique. Specifically, after an initial introduction with 2 cases performed in 2022, the case volume significantly increased over time (2 cases in 2023, 23 in 2024, 16 in 2025, and 5 in the first quarter of 2026).

Of these 48 patients, 28 underwent hip hemiarthroplasty, 4 underwent total hip arthroplasty following a hip fracture, and 16 underwent elective total hip arthroplasty. The median age of the sample was 83.5 years [IQR: 68.7–87.2], with a marked female predominance (66.7%). Regarding preoperative risk, the majority of patients were classified as ASA II (58.3%) or ASA III (22.9%), while 12.5% had an ASA I score.

The median time elapsed from the start of anesthesia to surgical incision (anesthetic preparation time) was 55 min [IQR: 50–76.2]. The median duration of surgery was 1.42 h (85 min) [IQR: 74–110].

It is important to note that patient preparation in our institution commonly begins before the preceding surgery has finished. The “Anesthestic Preparation Time” encompasses the entire preoperative pathway in the holding area, including patient positioning, standard monitoring, premedication, block execution, transfer to the operating room, final surgical positioning, and sterile draping until surgical incision.

In this case series, no patients needed further intraoperative analgesic or conversion to another type of anesthesia.

### 3.2. Efficacy and Safety

The SAFE technique, combined with spontaneous breathing sedation, was highly successful. Surgery was completed without the need for conversion to GA in all cases.

Postoperatively, all patients demonstrated the absence of motor blockade in the ankle, knee, and hip. No major immediate complications, such as Local Anesthetic Systemic Toxicity (LAST), allergic reactions, or accidental vascular punctures, were recorded.

Postoperative recovery room (RR) transit times were consistently documented for 35 out of the 48 patients. In this subset, the median length of stay in the RR was remarkably brief, standing at 40 min [IQR: 34.0–67.5]. It should be noted that this short permanence is primarily driven by the institutional logistical requirement of awaiting the final postoperative X-ray prior to ward discharge, rather than by a clinical need for prolonged anesthetic monitoring or physiological stabilization. This further reflects the rapid patient emergence and the immediate readiness for ward transfer facilitated by this specific anesthetic approach.

Four patients received a dose of rescue opioids in the recovery room before their return to the ward.

Additionally, surgeons subjectively noted minimal intraoperative blood loss (although this parameter was not formally quantified), due to the PVI-like (peripheral vasoconstrictor injection) effect of the SAFE technique.

## 4. Discussion

This study evaluated the clinical viability of the SAFE technique—combining A-PENG, PONG, and LIA under light sedation—as a primary anesthetic and analgesic strategy for hip surgery. Although initially implemented to mitigate risks in frail, elderly patients undergoing trauma surgery, our clinical experience demonstrates that this approach is equally effective and highly advantageous for younger patients undergoing elective total hip arthroplasty. Our findings demonstrate the successful completion of both hip hemiarthroplasties and total hip replacements without resorting to spinal anesthesia (SA) or general anesthesia (GA), while ensuring excellent postoperative pain control with standard multimodal analgesia and the complete preservation of motor function.

The literature extensively documents the challenges associated with anesthetizing frail elderly patients. Standard spinal anesthesia, while avoiding airway instrumentation, frequently triggers significant hemodynamic shifts [5]. Furthermore, the lack of a single peripheral nerve block capable of covering the entire surgical field necessitates combined approaches [14].

Our protocol builds upon the PENG block described by Girón-Arango et al., which effectively targets the anterior hip capsule while sparing the quadriceps muscle [10]. Because the posterior capsule is not covered by the PENG block, surgical anesthesia strictly requires a posterior blockade. Therefore, we utilized the PONG block—a variation in the deep posterior gluteal compartment block described by Vermeylen et al. [15]—targeting the fascial plane beneath the quadratus femoris muscle. This strategy avoids direct involvement of the sciatic nerve, thereby preventing motor block of the lower limb.

The results obtained perfectly align with current ERAS (Enhanced Recovery After Surgery) principles, extending their proven benefits to all treated age groups. By entirely sparing motor function, 100% of the patients maintained complete motor capacity, satisfying a primary prerequisite for immediate limb mobilization [8]. Furthermore, postoperative pain levels remained consistently minimal, allowing for low opioid consumption during the short-term recovery period. This limitation of opioid use is of crucial clinical importance not only in the geriatric population—where these drugs represent a primary trigger for postoperative delirium, nausea, and delayed functional recovery—but also in younger elective patients, facilitating an extremely rapid fast-tracking process and the concrete possibility of early discharge.

From a strictly technical standpoint, the procedure proved to be safe and reproducible. The non-conventional A-PENG approach (parallel to the femoral neck) proved particularly advantageous in patients with central obesity and remains feasible even in the lateral decubitus position, if necessary. However, it must be emphasized that performing these advanced ultrasound-guided fascial blocks requires an appropriate learning curve to ensure the precise deposition of the local anesthetic and to prevent unintended spread to the femoral or sciatic nerves, which could result in transient motor deficits.

We acknowledge the inherent limitations of this study. The retrospective design, the single-center nature of the cohort, and the relatively small sample size intrinsically limit the statistical power and the generalizability of the findings. Additionally, the lack of a formal control group prevents a direct and rigorous statistical comparison with current standards of care (spinal or general anesthesia). Finally, although the operators uniformly reported excellent intraoperative hemodynamic stability and a noticeable reduction in blood loss (likely attributable to the vasoconstrictive action of the epinephrine added to the anesthetic mixture), these variables were not formally measured and recorded in the analyzed electronic dataset.

## 5. Conclusions

This single-center, retrospective case series demonstrates the preliminary clinical feasibility of the SAFE technique (A-PENG + PONG + LIA with light sedation) for hip hemiarthroplasty in elderly patients. The technique provided adequate surgical anesthesia and postoperative analgesia while avoiding spinal or general anesthesia. Furthermore, the complete clinical preservation of motor function suggests a possible alignment with ERAS fast-track protocols. However, these conclusions must be interpreted with caution. The lack of a control group, the convenience sampling, and the retrospective design limit the generalizability of the findings and preclude definitive claims of superiority. The promising opioid-sparing and motor-sparing benefits observed in this feasibility study require rigorous validation through prospective, randomized controlled trials. Specifically, future research could compare the SAFE protocol against optimized, low-dose spinal anesthesia regimens to definitively establish its comparative efficacy, safety profile, and definitive role within modern fast-track pathways.

## Data Availability

The data presented in this study are available on request from the corresponding author.

## Abbreviations

GA	General anesthesia
SA	Spinal anesthesia
SAFE	Subarachnoid-alternative Anaesthesia For Endoprosthesis
A-PENG	Anterior Pericapsular Nerve Group block
PONG	POsterior pericapsular nerve block
LIA	Local Infiltration Analgesia
RR	Recovery room
IQR	Interquartile ranges
ERAS	Enhanced Recovery After Surgery
RA	Regional anesthesia
FICB	Fascia Iliaca Compartment Block
FNB	Femoral nerve block
TCI	Target Controlled Infusion
NRS	Numeric Rating Scale
Google LLC	Gemini large language model environment
LAST	Local Anesthetic Systemic Toxicity
PVI-like	Peripheral vasoconstrictor injection
PACU	Intraoperative and Post-Anesthesia Care Unit
